# In Vitro Activity of Imipenem and Colistin against a Carbapenem-Resistant *Klebsiella pneumoniae* Isolate Coproducing SHV-31, CMY-2, and DHA-1

**DOI:** 10.1155/2015/568079

**Published:** 2015-05-03

**Authors:** Hung-Jen Tang, Yee-Huang Ku, Mei-Feng Lee, Yin-Ching Chuang, Wen-Liang Yu

**Affiliations:** ^1^Department of Internal Medicine, Chi Mei Medical Center, Tainan City 71004, Taiwan; ^2^Department of Health and Nutrition, Chia Nan University of Pharmacy and Science, Tainan City 71710, Taiwan; ^3^Division of Infectious Disease, Department of Internal Medicine, Chi Mei Medical Center-Liu Ying, Tainan City 73657, Taiwan; ^4^Department of Medical Research, Chi Mei Medical Center, Tainan City 71004, Taiwan; ^5^Department of Intensive Care Medicine, Chi Mei Medical Center, No. 901 Zhonghua Road, Yongkang District, Tainan City 71004, Taiwan; ^6^Department of Internal Medicine, School of Medicine, College of Medicine, Taipei Medical University, Taipei City 11042, Taiwan

## Abstract

We investigated the synergism of colistin and imipenem against a multidrug-resistant *K. pneumoniae* isolate which was recovered from a severe hip infection. PCR and DNA sequencing were used to characterize the outer membrane porin genes and the resistance genes mediating the common *β*-lactamases and carbapenemases. Synergism was evaluated by time-kill studies. The *bla*_SHV-31_, *bla*_CMY-2_, and *bla*_DHA-1_ were detected. Outer membrane porin genes analysis revealed loss of *ompK36* and frame-shift mutation of *ompK35*. The common carbapenemase genes were not found. Time-kill studies demonstrated that a combination of 1x MIC of colistin (2 mg/L) and 1x MIC of imipenem (8 mg/L) was synergistic and bactericidal but with inoculum effect. Bactericidal activity without inoculum effect was observed by concentration of 2x MIC of colistin alone or plus 2x MIC of imipenem. In conclusion, colistin plus imipenem could be an alternative option to treat carbapenem-resistant *K. pneumoniae* infections.

## 1. Introduction

The widespread multidrug resistant Enterobacteriaceae is challenging physicians to effectively treat nosocomial infections. Followed by the extensive use of broad-spectrum antibiotics, the plasmid-mediated extended-spectrum *β*-lactamases (ESBLs) and/or AmpC *β*-lactamases have limited the choice of antibiotics to carbapenems for therapy against serious infections [1, 2]. However, increased use of carbapenems may contribute to the rising occurrence of carbapenem-resistant Enterobacteriaceae [3, 4]. In an era with limited antimicrobial agents available to us, old potential candidate (such as colistin) has therefore commonly been used or recommended to combine with a carbapenem to treat serious carbapenem-resistant Enterobacteriaceae infections [5, 6]. We have reported the synergistic effect by a combination of colistin and tigecycline against an ESBL-producing* Klebsiella pneumoniae* urine isolate with resistance to ertapenem, imipenem, and meropenem. However, there was a concern of inoculum effect because colistin plus tigecycline achieved less bactericidal effect against the strain with higher inoculum density in the bacterial suspensions [7]. Similarly, we experienced a multidrug resistant* K. pneumoniae* designed Kp830 isolated from a patient with severe hip infection. The isolate was resistant to imipenem according to the breakpoints of 2012 CLSI [8]. The goals of the study were to investigate the mechanisms of resistance to carbapenems and to evaluate the potential synergism between colistin and imipenem.

## 2. Material and Methods

### 2.1. Bacterial Isolates

A 79-year-old diabetic man was referred to the hospital due to persistent wound discharge from the left hip for one month. The computed tomography showed left hip and thigh necrotizing fasciitis and left iliac muscle abscess. Both of the deep-seated abscess and blood cultures yielded multidrug resistant* K. pneumoniae* isolates with the same antibiogram, which was resistant to amikacin, ampicillin, cefazolin, cefuroxime, ceftazidime, ciprofloxacin, ertapenem, flomoxef, gentamicin, and piperacillin-tazobactam but was susceptible to imipenem, using standard disc diffusion test [8]. The organism was not eradicated and the patient died in acute renal failure, acute respiratory failure, and septic shock after 9-week hospitalization, despite of three times of surgical debridement and 7-week imipenem therapy. The last wound pus* K. pneumoniae* isolate became resistant to imipenem and was designed strain Kp830, which was subcultured and frozen at −70°C until being used in the study.

### 2.2. Antimicrobial Susceptibility Testing

Minimal inhibitory concentrations (MICs) for imipenem (Merk, Sharp & Dohme, West Point, PA, USA) and colistin sulphate (Sigma Chemical Company, St. Louis, MO, USA) were determined by standard agar dilution method according to CLSI [9].

There is no CLSI recommendation for colistin susceptible breakpoints against Enterobacteriaceae [8]. According to the British Society for Antimicrobial Chemotherapy Working Party on Susceptibility Testing, the susceptible MIC breakpoint for colistin against Enterobacteriaceae is ≦4 *μ*g/mL and should be considered resistant if MIC >4 *μ*g/mL [10]. We applied the British Society MIC breakpoints to our results.

### 2.3. Phenotypic Methods for Detection of *β*-Lactamases

The ESBL production was interpreted by the phenotypic confirmatory test according to CLSI disk diffusion method [8].* Escherichia coli* ATCC 25922 and* K. pneumoniae* ATCC 700603 were used as the negative and positive control, respectively. An increase of ≧5 mm between the growth-inhibitory zone diameter of either cefotaxime or ceftazidime tested in combination with clavulanate and its zone diameter when tested alone is interpreted as positive ESBL phenotype.

In the present study, 3-aminophenylboronic acid (APB) (Sigma-Aldrich, Steinheim, Germany) was used in the disk potentiation test and double-disk synergy test for the identification of class C *β*-lactamase production. The enlargement and discernible expansion of diameter of the growth-inhibitory zone were observed in plasmid-mediated class C *β*-lactamase producing bacteria [11]. The modified Hodge test (MHT) was used to detect carbapenemase production when the test isolate produced the enzyme and allowed growth of a carbapenem susceptible strain (*E. coli *ATCC 25922) towards a carbapenem disk [8]. The modified MHT is limited by unknown sensitivity and specificity for detecting low-level metallo-*β*-lactamase production [8].

### 2.4. Detection of *β*-Lactamase Genes

Plasmid DNA was extracted as templates and polymerase chain reaction (PCR) was used to amplify *bla*_CTX-M_, *bla*_TEM_, *bla*_IMI_,* bla*_GES_,* bla*_IMP_,* bla*_VIM_,* bla*_KPC_,* bla*_OXA_ (OXA-23, OXA-24, OXA-48, and OXA-58), and* bla*_NDM_ using specific primers as previously published [3, 12, 13]. For AmpC genes, the following primers were used: (a) CMY-2-forward (TTT TCA AGA ATG CGC CAG GC), CMY-2-reverse (CTG CTG CTG ACA GCC TCT TT); (b) DHA-1-forward (CTG ATG AAA AAA TCG TTA TC) and DHA-1-reverse (ATT CCA GTG CAC TCA AAA TA). For SHV genes, the following primers were used: (a) SHV-forward (GAT CCA CTA TCG CCA GCA GG) and SHV-reverse (ACC ACA ATG CGC TCT GC TTT G); (b) SHV-12-forward (ATG CGT TAT ATT CGC CTG TG) and SHV-12-reverse (TTA GCG TTG CCA GTG CTC G). Amplicons were purified with PCR clean-up kits (Roche Diagnostics, GmbH, Penzberg, Germany) and sequenced on an ABI PRISM 3730 sequencer analyzer (Applied Biosystems, Foster City, CA, USA).

### 2.5. Detection of Outer Membrane Porin Genes

Outer membrane porin-associated genes (*ompK35* and* ompK36*) were screened by using PCR assay as previously described [14]. The PCR amplicons were sequenced and analyzed with the BLAST program.

### 2.6. Time-Kill Assay

Model time-kill curves were determined by plotting mean colony counts (log10 CFU per milliliter) from each model versus time. All model simulations were conducted over 48 h and were performed in duplicate to ensure reproducibility. The 1x MIC and 2x MIC for drugs concentration of imipenem and colistin alone or combination of both drugs were investigated in time-kill studies. Approximately 2 × 10^5^ CFU/mL (standard inoculum) and 1.6 × 10^6^ CFU/mL (higher inoculum) were used at baseline. Serial samples (baseline, 2, 4, 6, 8, 12, 24, 28, 32, and 48 h) were obtained for 48 hours. Total bacterial populations were quantified by serial dilution. Bactericidal activity was defined as a ≧3log⁡_10_⁡ CFU/mL decrease in the viable cell counts within 24 hours with respect to the original inoculum. Synergistic effect was defined as a ≧2log⁡_10_⁡ CFU/mL decrease in the viable cell counts compared to the most active drug. Inoculum effect was defined as a decreasing antibiotic efficacy with high inoculum. The lower limit of detection was 1log⁡_10_⁡ CFU/mL.

## 3. Results

The MICs of imipenem and colistin for the strain Kp830 were 8 mg/L and 2 mg/L, respectively. The 2012 CLSI recommended imipenem MIC breakpoints as susceptible, ≦1 mg/L, and resistant, ≧4 mg/L [8].

**Figure 1:**
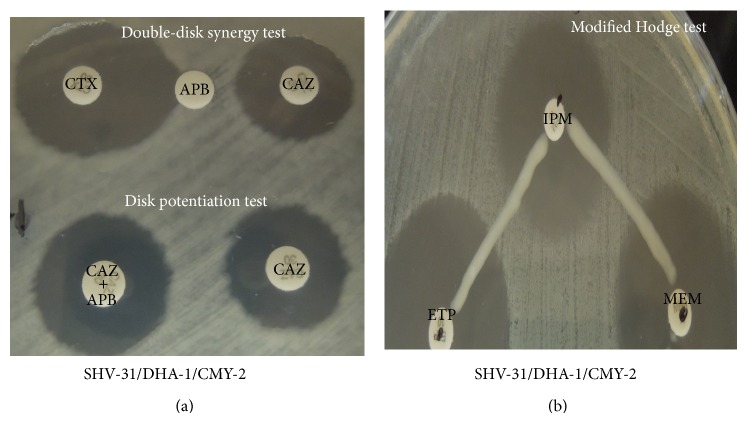
Phenotypic disc confirmatory tests. (a) Double-disk synergy test and disk potentiation test using cefotaxime (CTX) disk and ceftazidime (CAZ) disk are confirming the production of class C *β*-lactamases which were inhibited by APB. (b) The modified Hodge test is showing negative results for carbapenemase production, when the test isolate (Kp830) grew towards three carbapenem disks, including imipenem (IPM), meropenem (MEM), and ertapenem (ETP).

The carbapenemase phenotype of the strain Kp830 was negative, whereas the class C *β*-lactamase phenotype was positive (Figure 1). Further detection of the *β*-lactamase genes for the plasmid DNA extract from the strain Kp830 confirmed the presence of plasmid-mediated *bla*_SHV-31_ (GenBank accession number, KC880337), *bla*_CMY-2_, and *bla*_DHA-1_. The amino acid sequence of SHV-31 differs from SHV-1 by two mutations, namely, L35Q and E240K, and differs from SHV-12 by one mutation (G134S). PCR results for other *β*-lactamase genes were all negative. PCR for the outer membrane porin genes and DNA sequencing analysis revealed loss of porin* ompK36* gene and frame-shift mutation in* ompK35* gene (100% identity to GenBank accession number GU945370), similar to previous reports [15, 16].

**Figure 2:**
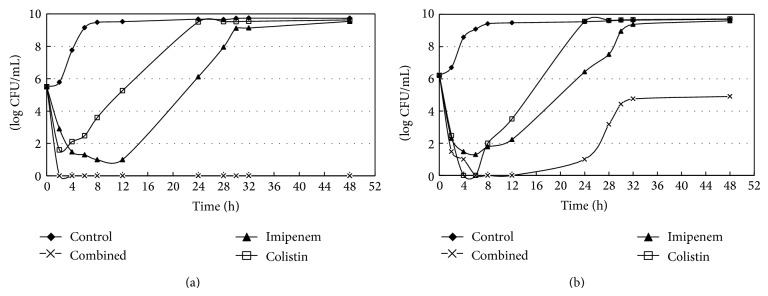
Survival curves of Kp830 (a) 1x MIC, in standard inoculum, and (b) 1x MIC, in high inoculums. All models with duplicate performance demonstrated similar bactericidal kill (duplicate data not shown).

**Figure 3:**
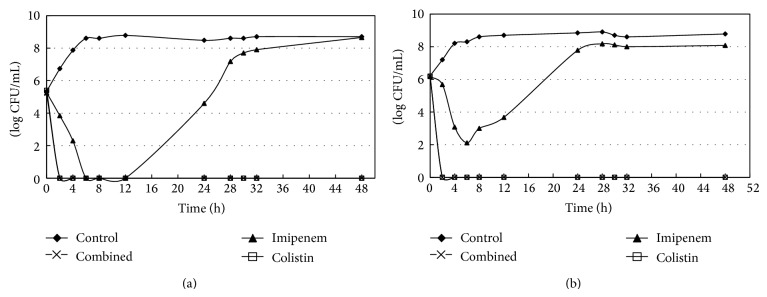
Survival curves of Kp830 (a) 2x MIC, in standard inoculums, and (b) 2x MIC, in high inoculums. All models with duplicate performance demonstrated similar bactericidal kill (duplicate data not shown).

Data from the time-kill studies in different inocula and multiples of MIC are shown in Figure 2(a) (standard inoculum, 1x MIC), Figure 2(b) (high inoculum, 1x MIC), Figure 3(a) (standard inoculum, 2x MIC), and Figure 3(b) (high inoculum, 2x MIC). The initial bactericidal activities of colistin and imipenem were attenuated by regrowth after 6–8 hours of antibiotic exposure at 1x MIC with different inocula (Figures 2(a) and 2(b)). Colistin showed excellent bactericidal activity against Kp830 strain at 2x MICs with different inocula (no inoculum effect), which began at 2 h after inoculations and has sustained for 48 h (Figures 3(a) and 3(b)). For 2x MICs of imipenem, inoculum effect and delayed regrowth after 12-hour exposure were also observed, even the initial bactericidal activity producing a decrease >3log⁡_10_⁡ CFU/mL after a 6-hour exposure.

Synergism was observed in the combination of colistin and imipenem each at 1x MIC with standard inoculum (Figure 2(a)). However, combination of colistin with imipenem each at 1x MIC for high-inoculum bacterial densities demonstrated early bactericidal effect with delayed regrowth after 24-hour exposure (Figure 2(b)). Due to excellent bactericidal activity of colistin, synergism of colistin and imipenem was not observed at 2x MICs with different inocula (Figures 3(a) and 3(b)).

## 4. Discussion

The coproduction of ESBL (SHV-31) and AmpC *β*-lactamases (CMY-2 and DHA-1) may confer resistance of strain Kp830 to multiple drugs, such as ceftazidime, flomoxef, and piperacillin-tazobactam. The SHV-31 differs from SHV-1 by the two mutations, namely, L35Q and E240K, which was first reported in The Netherlands [15]. Although there might be a chance of false-negative result for modified MHT, we extensively surveyed the common carbapenemase genes and all the results were negative. The negative modified MHT in Kp830 may correspond to the bactericidal activity of 1x MIC and 2x MIC imipenem, even though the strain was resistant to imipenem according MIC breakpoints. The reasons of carbapenem resistance could be explained by the OmpK36 and/or OmpK35 defects, which have represented the major mechanism for the development of carbapenem resistance in the ESBL- and/or AmpC-producing* K. pneumoniae* isolates in Taiwan [16–18]. Our case highlighted that prolonged imipenem use may be associated with* ompK35* gene mutation and* ompK36* gene loss on ESBL-producing* K. pneumoniae *and led to emergence of carbapenem resistance.

Lai et al. reported an increasing trend with a prevalence of about 2.5% of carbapenem-nonsusceptible Enterobacteriaceae in Taiwan [19]. In that study, carbapenem-based therapy (most commonly combined with amikacin) still had a good outcome with a 90% clinical success rate. Because our strain Kp830 was resistant to amikacin but susceptible to colistin, there is a need to document the in vitro efficacy of carbapenem plus colistin. The time-killing data might suggest high-dosage colistin alone or in combination with imipenem to treat serious carbapenem-resistant* K. pneumoniae* infections. Souli et al. reported that synergy of colistin and imipenem was observed only against isolates exhibiting susceptibility or low-level resistance to colistin [20]. The current study revealed that synergy of colistin and imipenem was observed only with 1x MICs of both drugs against the isolate with normal inoculum but there was an inoculum effect. The inoculum phenomenon of declining antibiotic efficacy may lead to treatment failure for serious infections with high bacterial population in the lesions, such as endocarditis, meningitis, septic arthritis, osteomyelitis, abscesses, and other deep-seated infections [21].

In conclusion, an imipenem-resistant* K. pneumoniae* with outer membrane porin defect as well as coproducing SHV-31, CMY-2, and DHA-1 emerged from a prolonged course of imipenem therapy. A high-dosage colistin alone or in combination with imipenem may be considered an alternative option to treat the carbapenem-resistant* K. pneumoniae* infections.
